# Mechanism of Interaction of Al^3+^ with the Proteins Composition of Photosystem II

**DOI:** 10.1371/journal.pone.0120876

**Published:** 2015-03-25

**Authors:** Imed Hasni, Hnia Yaakoubi, Saber Hamdani, Heidar-Ali Tajmir-Riahi, Robert Carpentier

**Affiliations:** 1 Research Group in Plant Biology, Department of Chemistry, Biochemistry and Physics, University of Quebec at Trois-Rivieres, Trois-Rivieres, Quebec, Canada; 2 Plant Systems Biology Group, Partner Institute of Computational Biology, Chinese Academy of Sciences, Shanghai, China; University of Hyderabad, INDIA

## Abstract

The inhibitory effect of Al3+on photosystem II (PSII) electron transport was investigated using several biophysical and biochemical techniques such as oxygen evolution, chlorophyll fluorescence induction and emission, SDS-polyacrylamide and native green gel electrophoresis, and FTIR spectroscopy. In order to understand the mechanism of its inhibitory action, we have analyzed the interaction of this toxic cation with proteins subunits of PSII submembrane fractions isolated from spinach. Our results show that Al^ 3+^, especially above 3 mM, strongly inhibits oxygen evolution and affects the advancement of the S states of the Mn_4_O_5_Ca cluster. This inhibition was due to the release of the extrinsic polypeptides and the disorganization of the Mn4O5Ca cluster associated with the oxygen evolving complex (OEC) of PSII. This fact was accompanied by a significant decline of maximum quantum yield of PSII (F_v_/F_m_) together with a strong damping of the chlorophyll a fluorescence induction. The energy transfer from light harvesting antenna to reaction centers of PSII was impaired following the alteration of the light harvesting complex of photosystem II (LHCII). The latter result was revealed by the drop of chlorophyll fluorescence emission spectra at low temperature (77 K), increase of F_0_ and confirmed by the native green gel electrophoresis. FTIR measurements indicated that the interaction of Al^ 3+^ with the intrinsic and extrinsic polypeptides of PSII induces major alterations of the protein secondary structure leading to conformational changes. This was reflected by a major reduction of α-helix with an increase of β-sheet and random coil structures in Al^ 3+^-PSII complexes. These structural changes are closely related with the functional alteration of PSII activity revealed by the inhibition of the electron transport chain of PSII.

## Introduction

In higher plants, oxygenic photosynthesis is considered as one of fundamental processes of life that transforms light into chemical energy. This process takes place in photosystem II (PSII) embedded in the thylakoid membranes of the chloroplast. PSII is a multisubunit membrane protein complex, composed of more than 25 intrinsic and extrinsic proteins, that catalyzes the oxidation of water and the reduction of plastoquinone (PQ) [1–10]. The intrinsic proteins include several transmembrane subunits such as D1, D2, CP43, CP47 and the α and β subunits of cytochrome b559 which constitute the reaction centre (RC) of PSII [11–13]. It has been shown that the transmembrane intrinsic polypeptides are rich in α-helices as they contain at least 29 different transmembrane α-helices [14]. Deletion of these intrinsic proteins leads to the complete loss of functional PSII and assembly [15].

On the luminal side of the thylakoid membrane, three extrinsic proteins associated with PSII core participate intensively in the oxygen evolving activity: PsbO, PsbP and PsbQ with apparent molecular masses of 33, 23 and 17 kDa, respectively [16–19]. These extrinsic proteins are associated with the inorganic Mn_4_O_5_Ca cluster to form the oxygen evolving complex (OEC) which is considered as the heart of the water-oxidizing machinery of photosynthesis [20–22]. The Mn_4_O_5_Ca cluster contains four Mn ions, one Ca^2+^, and five oxo and is bounded by two Cl^−^ ions that act as indispensable cofactors to catalyze the oxygen evolving reaction [21–23]. The PsbO protein is known as the “manganese stabilizing protein” (MSP) given its important role in the stabilization of the Mn_4_O_5_Ca cluster. It has been known that its depletion significantly retards the S states transition in the Mn_4_O_5_Ca cluster [17, 18, 24]. PsbP and PsbQ proteins seem to modulate the functional roles of Ca^2+^ and Cl^−^ in oxygen evolution [17, 25–27].

Moreover, the OEC is associated with intrinsic transmembrane proteins to form the PSII heterodimeric core that binds the redox-active cofactors involved in electron transfer of PSII [28]. Roose et al. (2010) [29] suggested that the removal of the PsbP and PsbQ extrinsic polypeptides may induce transmembrane alterations in the structure of PSII complex leading to disruption of the Q_A_ and/or Q_B_ sites or modification of the plastoquinone-plastoquinol exchange channel.

The PSII RC is surrounded by two systems of pigment-protein complexes responsible for the light harvesting: the peripheral antenna called the light harvesting complex of PSII (LHCII) and the inner antenna located close to the RC. LHCII is the most abundant membrane protein which binds chlorophyll (Chl) *a* and *b*. It has been considered as a major peripheral antenna complex able to absorb light energy and transfer it to the RC of PSII via the inner antenna [30–33]. This latter antenna includes the CP47 and CP43 proteins which connect the PSII RC to the minor antenna proteins CP29, CP26 and CP24 and LHCII in order to perform transfer of excitation energy from LHCII to RC [2, 5, 34, 35].

Roose et al. (2007) [19] and Boekema et al. (2000) [36] have determined the relation between different subunits of PSII. They claimed that the release of the extrinsic proteins associated with the OEC affects the intrinsic core components of PSII. Moreover, they suggested that the removal of the two extrinsic polypeptides PsbP and PsbQ (of 23 and 17 kDa) may change the peripheral antenna proteins positions. Also, it has been proposed that the removal of the third extrinsic polypeptides PsbO (of 33 kDa) induce a destabilization in the dimeric structure of PSII leading to the conformational changes, which may be important for the assembly and disassembly of the PSII complex [19, 36].

The photochemical events are initiated by the absorption of the photons by the antenna complexes, especially the LHCII. This excitation energy is rapidly transferred by CP43 and CP47 toward the RC chlorophyll *a* (P680) leading to the formation of the excited state P680*. This state of P680 (P680*) is followed by a charge separation to reduce pheophytin (Pheo) allowing the formation of the P680^+^Pheo^-^ pair. On the acceptor side, one electron is transferred from the reduced Pheo^-^ to the primary quinone of PSII, Q_A_ and then to the secondary quinone Q_B_. Following two successive electrons Q_B_ becomes fully reduced and can accept two protons to form the plastoquinol molecule (PQH_2_). In parallel, the P680^+^ radical is rapidly reduced by a redox active tyrosine Tyr Z (Tyrosine 161 of D1 subunit) that extracts electrons from the Mn_4_O_5_Ca cluster of the OEC. The Mn_4_O_5_Ca cluster is characterized by five distinct oxidized states (S_0_, S_1_, S_2_, S_3_, and S_4_), known as S states where S_1_ is considered as the dark stable state of the OEC. In this site, water oxidation reaction is performed through the cycle of advancement of S states and four successive quanta of excitation are required for the transition from S_0_→ S_1_ → S_2_ → S_3_ → (S_4_) → S_0_. At the end of the S state cycle, especially the transition from S_4_ to S_0_ is accompanied by oxidation of two water molecules and the formation of oxygen molecule.

PSII complex has been considered to be the major target of several toxic metal cations [37–42]. Among these, Al^3+^, the solubilized toxic form of aluminum in acid soils, represents one of the major environmental stresses [43–47]. Owing to its abundance, aluminum, the third most common element in the earth’s crust, acts as a highly toxic non-essential element for plants under its cationic form, Al^3+^ [43, 44]. In several plant species, Al^3+^ is absorbed by roots and translocated to the leaf tissues where it is accumulated especially in the chloroplast [48]. At this level, the presence of Al^3+^ affects the photosynthesis process [45–49]. Several studies have showed that this trivalent cation (Al^3+^) inhibits photosynthetic electron transport in PSII and affects the PSII RC, causing the impairment of PSII activity [47–51].

Recent study has demonstrated that Al^3+^ interacts with different sites of PSII in isolated thylakoid membranes of spinach (*Spinacia oleracea L*.), leading to inhibition of oxygen evolution [42]. This inhibition was associated with the destabilization of the OEC including the disorganization of the Mn_4_O_5_Ca cluster at the donor side. Similar studies have associated this destabilization to the interaction of several cations such as Cd^2+^, Cu^2+^, Hg^2+^, Ni^2+^, Pb^2+^ and Zn^2+^ with the luminal side of PSII causing the release of the three extrinsic polypeptides of 17, 23 and 33 kDa associated with the OEC [37, 39, 41, 52, 53]. Further, Yruela et al. (2000) [53] have reported that the release of the OEC proteins was accompanied by a destabilization and liberation of inner antenna proteins CP47 and CP43 of PSII in the presence of high concentration of Cu^2+^. Also, Fagioni et al. (2009) [54] have showed that the Cd and Cu alter the structure and organization of the LHCII complex leading to a change in LHCII protein conformation.

In addition, Hasni et al. (2013) [42] have demonstrated that Al^3+^ induces an inhibition of electron transfer between Tyr Z and P680 causing the reduction of P680^+^ form. Thus, this may cause an impairment of Q_B_ reduction by P680 leading to a loss of electron transport through acceptor side of PSII which induces a decrease of the maximal fluorescence yield. Also, numerous studies have shown that the maximum quantum efficiency of PSII (F_v_/F_m_) decreases under aluminum stress [45, 48, 55, 56]. Furthermore, Li et al. (2012) [48] have suggested that the inhibition of PSII activity in tobacco leaves subjected to aluminum stress may be due to the reaction of Al^3+^ with the non-heme iron located between Q_A_ and Q_B_. Nahar et al. (1997) [57] have reported that the interaction of Ga^3+^ and Al^3+^, at high concentrations, with proteins of PSII causes a major conformational change of protein secondary structure. However, in spite of these studies, to our knowledge little information is available regarding the mechanisms of interaction of Al^3+^ with PSII complex and its effects on the structural change of proteins, proteins composition, and functionality of PSII complex.

In order to understand the mechanism of inhibitory action of Al^3+^ in PSII by focusing on its effect on the relation between the secondary structure of PSII proteins and the functional activity of PSII complex, we have analyzed the interaction of this cation at various concentrations with intrinsic and extrinsic protein subunits of PSII submembrane fractions isolated from spinach. For this purpose, different biophysical and biochemical techniques have been used. Water oxidation, S states transitions, Chl fluorescence induction and emission, electron transfer at both sides of PSII, polypeptides composition of both OEC and LHCII were affected, and structural changes of PSII complex have been noted.

## Materials and Methods

### Thylakoid membrane preparation

Thylakoid membranes were isolated from fresh spinach (*Spinacia oleracea* L.) leaves, obtained from a local market (IGA, Trois-Rivières, Qc, Canada), according to Joly et al. (2005) [58] and the Chl content was determined as described in Porra et al. (1989) [59].

### Isolation of PSII submembrane fractions

PSII submembrane fractions were isolated from thylakoid membranes as described elsewhere [60] with minor modifications. Following incubation of isolated thylakoid membranes for 90 min in the dark at ice-cold temperature, Triton X-100 was added with gently shaking for 1 min to obtain a final concentration of 1 mg Chl.ml^-1^. The latter solution was incubated 1 min in the dark and centrifuged for 4 min at 600 x g. The resulting supernatants were centrifuged for 15 min at 35300 x g. The pellet was suspended in a buffer containing 20 mM Mes-NaOH (pH 6.2), 15 mM NaCl, 10 mM MgCl_2_, and 400 mM sucrose and centrifuged at 4960 x g for 4 min. Collected supernatants were centrifuged at 35300 x g for 15 min and their pellets were suspended in the same buffer. This homogenate of pellets was centrifuged for 15 min at 35300 x g. At the latest step, the new pellet obtained was suspended in the same buffer and the Chl content was calculated following the procedure described in Porra et al. (1989) [59].

### Oxygen evolution activity measurements

The rate of oxygen evolution of PSII submembrane fractions samples was performed with Clark type electrode at 24°C under continuous saturating white light using Oxylab system (Hansatech Instrument, Norfolk, England). The assay medium contained 20 mM MES-NaOH (pH 6.2), 1 mM NaCl, 0.5 mM MgCl_2_, 0.35 mM DCBQ (2.5-dichlorobenzoquinone) as PSII electron acceptor, 25 μg Chl.ml^-1^ of PSII submembrane fractions, and the specified Al^3+^ concentrations added as Al_2_(SO_4_)_3_.

Oxygen flash yields of isolated thylakoid membranes were recorded at room temperature by a laboratory built polarographic oxygen rate electrode described in Zeinalov (2002) [61]. The sample at 200 μg.ml^-1^ of Chl concentration was incubated 3 minutes in the dark before measurements. At each measurement, the dark adapted sample was illuminated by a train of 12 saturating (4J) single turnover flashes (10 μs). The assay medium contained 40 mM Hepes-NaOH (pH 7.6), 10 mM NaCl, 5 mM MgCl_2_, 400 mM sucrose, and the specified concentrations of Al^3+^. The Oxygen yields of the 12 flashes and their parameters were estimated using developed analytical solution for the fitting of experimental data as described previously in Messinger et al. (1997) [62] based on extended Kok model [63].

### SDS-polyacrylamide gel electrophoresis

PSII submembrane proteins were separated by polyacrylamide gel electrophoresis (SDS-PAGE) using miniature slab gels (Bio-Rad Laboratories, Hercules, California) containing 13% acrylamide and 6 M urea according to Laemmli (1970) [64]. Samples of PSII submembrane fractions at 100 μg Chl.ml^-1^ were treated with different concentrations of Al^3+^, incubated for 5 min at room temperature in the dark and centrifuged at 12400 rpm for 5 min in an Eppendorf microcentrifuge. The pellets were washed twice in 20 mM Mes-NaOH (pH 6.2) centrifuged at 12400 rpm for 5 min and then used for polypeptides separation in the gel. The Tris-alkali extraction of the 17, 23 and 33 kDa polypeptides was carried out basically as described in Nakatani (1984) [65] and then concentrated against sucrose using Spectra/Por Molecularporous membranes (Spectrum Laboratories, Inc., Rancho Dominguez, CA, USA). 10 μl of the different samples of PSII submembrane fractions, treated with Al^3+^ concentrations and the Tris-alkali extraction, were loaded per lane onto the gel. Finally, SDS-polyacrylamide gels containing separated polypeptides were stained with Coomassie brilliant blue and analyzed with the Gel-Doc 2000 system (Bio-Rad Laboratories, Hercules, CA, USA).

### Chl fluorescence induction

Chl fluorescence induction (FI) measurements were carried out at room temperature using Plant Efficiency Analyser (Hansatech, Kings Lynn, Norfolk, UK). The assay medium contained 20 mM MES-NaOH (pH 6.2), 15 mM NaCl, 10 mM MgCl_2_, 400 mM sucrose, PSII submembrane fractions at 25 μg Chl.ml^-1^ and the specified concentrations of Al^3+^. Samples were adapted for 1 min in the dark and then excited with saturating red actinic light (peaking at 655 nm and intensity of 3000 μmol photons m^-2^ s^-1^) provided by light emitting diodes. As the fluorescence signal during the first 40 μs is ascribed to artifacts due to delay in response time of the instrument, these data were not included in the analysis of FI traces. The signal at 40 μs is taken as F_0_, the initial fluorescence intensity. Variable fluorescence, F_v_ (the difference between F_0_ and the maximal fluorescence, F_m_ in dark adapted samples) was used to calculate the F_v_/F_m_ and F_v_/F_0_ ratios.

### Low temperature (77 K) chlorophyll fluorescence measurements

Fluorescence emission spectra from isolated thylakoid membranes were measured at 77 K using the Perkin-Elmer LS55 spectrofluorimeter equipped with an R928 red-sensitive photomultiplier (Woodbridge, ON, Canada). The assay medium contained 20 mM Hepes-NaOH (pH 7.6), 10 mM NaCl, 2 mM MgCl_2_, 20 mM KCl, 400 mM sucrose, 5 μg Chl.ml^-1^ and the specified Al^3+^ concentrations with the presence of 60% glycerol. Chl fluorescence was excited at 436 nm and emission spectral was detected from 650 to 800 nm as described by Rajagopal et al. (2002) [66]. The excitation and emission spectral widths were fixed at 5 and 2.5 nm, respectively. Emission spectra were corrected according to the photomultiplier sensitivity using the correction factor spectrum provided by Perkin-Elmer. The spectra were normalized at 732 nm.

### Native green gel electrophoresis

Separation of different chlorophyll-protein complexes of isolated thylakoid membranes was performed following the method described previously [67]. The samples of isolated thylakoid membranes were incubated in the dark for 5 min at different Al^3+^ concentrations and then centrifuged at 10000 rpm for 10 min at 4°C in an Eppendorf microcentrifuge. The pellets were washed the first time in ice cold 2 mM Tris-maleate buffer (pH 7.0), then centrifuged at 12500 rpm for 15 min at 4°C. The resulted pellets were washed second time in ice cold 2 mM Tris-maleate-10% glycerol buffer (pH 7.0), then centrifuged at 12500 rpm for 15 min and finally solubilized for 30 min on ice in a buffer solution contained 0.45% (w/v) octyl glucoside, 0.45% (w/v) decyl maltopyroside, 0.1% (w/v) lithium dodecyl sulfate, 10% (v/v) glycerol and 2 mM Tris-maleate (pH 7.0) to adjust the ratio of total non-ionic detergents to Chl at 20:1 (w/w). The unsolubilized fragments were removed by centrifugation at 11000 rpm for 5 min and the supernatant obtained was loaded onto a 5% stacking polyacrylamide gel. Chlorophyll-protein complexes were resolved on a 12% separating polyacrylamide gel. Gels were run at 4°C for 1–2 h at a constant current of 10 mA and then photographed.

### FTIR spectroscopic measurements

Infrared spectra measurements were performed using the FTIR spectrometer (Impact 420 model), equipped with deuterated triglycine sulphate (DTGS) detector and KBr beam splitter, using AgBr windows. The concentration of PSII submembrane fractions was 1 mg Chl.ml^-1^. Samples were prepared by addition of Al^3+^ to the PSII submembrane fractions at concentrations of 1, 2, 3, 4 and 5 mM. Spectra were collected after 4 h incubation of PSII with Al^3+^ concentrations at room temperature in the dark using hydrated films. Interferograms were accumulated over the spectral range 4000–600 cm^-1^ with a nominal resolution of 4 cm^-1^ and 100 scans.

### Analysis of PSII protein secondary structure

Analysis of the secondary structure of PSII proteins with the presence or not of Al^3+^ concentrations was carried out as described in Ahmed et al. (1995) [68]. For determination of secondary structure of PSII proteins, the shape of the amide I band, located around 1660–1650 cm^-1^ was used. Spectral analysis was performed using the GRAMS/AI Version 7.01 software of the Galactic Industries Corporation. The FTIR spectra were smoothed and their baselines were corrected automatically. Thus the root-mean square (rms) noise of every spectrum was calculated. By means of the second derivative in the spectral region 1600–1700 cm^-1^ five major peaks for free PSII and their Al^3+^ complexes were resolved. The spectral region was deconvoluted by the curve-fitting method following the Levenberg-Marquadt algorithm, and the peaks corresponds to α-helix (1654–1660 cm^-1^), β-sheet (1637–1614 cm^-1^), turn (1678–1670 cm^-1^), random coil (1648–1638 cm^-1^) and β-antiparallel (1691–1680 cm^-1^) were adjusted. The area of all the component bands was measured with the Gaussian function, and then summed up and divided by the total area [69].

## Results

### Oxygen evolution

**Fig 1:**
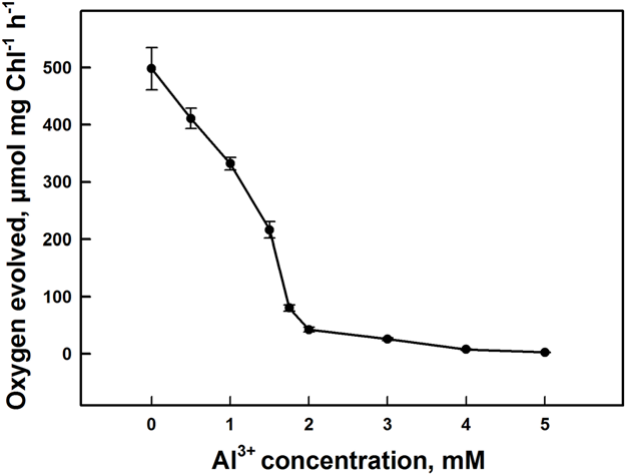
Inhibition of oxygen evolution activity in PSII submembrane fractions under effect of different concentrations of Al^3+^. Each point represents the mean ± SD of nine independent measurements obtained from three different samples. Details are given in "Materials and methods" section.

Fig. 1 shows the effects of Al^3+^ on photosynthetic oxygen evolution activity in PSII submembrane fractions isolated from spinach. This parameter was measured with DCBQ as specific artificial electron acceptor for PSII in the presence or not of Al^3+^. Oxygen evolution activity decreased significantly with increasing aluminum cation concentration. A sharp 91% drop in the oxygen evolution occurred by the addition of 2 mM of Al^3+^ compared with the control. Above this concentration, the oxygen evolution decreased slightly until it reached 99% at 5 mM of Al^3+^. We note that the loss of oxygen evolution activity under Al^3+^ action was more pronounced in PSII submembrane fractions compared to thylakoid membranes [see 42]. This difference is likely due to a more accessible binding of Al^3+^ in PSII submembrane fractions than in thylakoid membranes due to their structural differences. In addition, PSII is considered a more simple and specific system without interference from other components of the thylakoid membrane.

**Fig 2:**
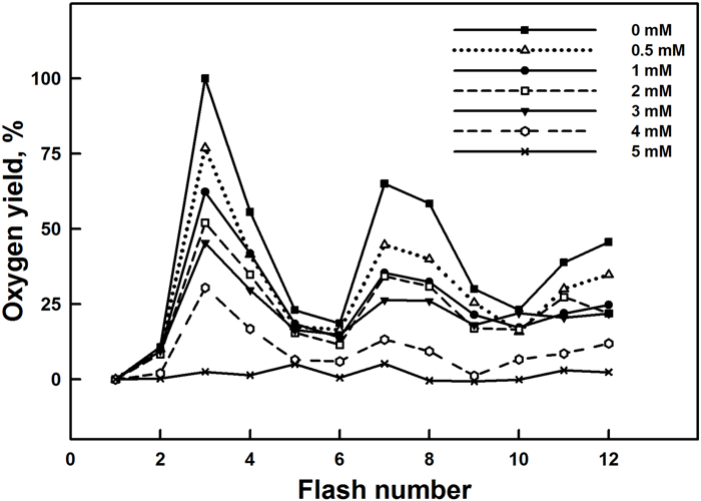
Effect of various Al^3+^ concentrations on the period of oscillation of the yield of oxygen evolution. All traces have been normalized to the third flash in the control sample. Each trace is the average of nine independent measurements with samples from three different batches.

Similar trends were observed during the measurements of flash-induced oxygen evolution of dark adapted isolated thylakoid membranes from spinach in Fig. 2. The flash-induced oxygen evolution patterns, for the control dark adapted thylakoid membranes, show a typical period of four oscillations with first maxima on the third flash. This periodicity is related to the advancement of the S states of the Mn_4_O_5_Ca cluster in the OEC that generates an oxygen molecule at the third flash when samples are excited after dark adaptation [63].

Addition of 0.5–5 mM Al^3+^ in thylakoid membranes induced a decline in the amplitudes of the flash-induced oxygen yields. This decline was accentuated with increasing concentrations of Al^3+^ and the oscillation pattern was also modified.

This effect was accompanied by modification of the parameters of oxygen yields such as misses (zero-step advance), hits (one-step advance) and double hits (double-step advance) determined according to the Kok’s model. Data shows an increase in the percentage of misses and double hits, and a decrease in the percentage of hits with increasing Al^3+^ concentrations (Table 1). This reflects the reduction and/or destruction of oxygen evolving complex at the donor side.

**Table 1:** Effect of the addition of Al^3+^ concentrations on the oxygen flash yields parameters for isolated thylakoid membranes treated with various concentrations of Al^3+^.

Parameters, %	Al3+ concentration, mM						
	0	0.5	1	1.5	2	3	4
**Misses (±2%)**	13.9	13.8	13.9	14.6	14.9	16.9	20.3
**Hits (±3%)**	84.9	84.3	83.5	82.7	82.1	79.3	75.8
**Double-Hits (±1%)**	1.3	2.0	2.5	2.7	3.0	3.7	3.8

The data are average ± SD from nine independent experiments.

### PSII polypeptide profile in SDS page electrophoresis

**Fig 3:**
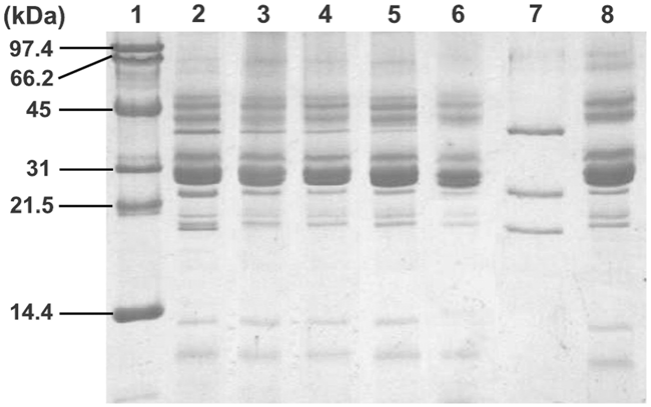
SDS-PAGE polypeptide profile of PSII submembrane fractions treated with different concentrations of Al^3+^. Lane 1, molecular weight standards; lane 2, control PSII; lane 3, 1 mM Al^3+^ treated PSII; lane 4, 2 mM Al^3+^ treated PSII; lane 5, 4 mM Al^3+^ treated PSII; lane 6, 5 mM Al^3+^ treated PSII; lane 7, supernatant of the Tris-alkali treated PSII; lane 8, Tris-alkali treated PSII. Numbers on the left side indicate the masses (in KDa) of molecular markers. See details in the "Materials and methods" section.

In order to get more information about the interaction of Al^3+^ with the polypeptides composition of PSII submembrane fractions, especially the extrinsic polypeptides associated with the OEC, we used the polyacrylamide gel electrophoresis. The polypeptide profile of PSII submembrane fractions after various treatments of Al^3+^ concentration is shown in Fig. 3. To identify the three extrinsic polypeptides of the OEC, we incubated PSII submembrane fractions with Tris-alkali (pH 9.2), a specific treatment that causes release of three extrinsic polypeptides of the OEC from their positions in PSII complex. In lane 7, the positions that correspond to the extrinsic polypeptides associated with the OEC are indicated by their specific molecular weight of 17, 23 and 33 kDa in the Tris-alkali supernatant fractions and were also used as reference added to molecular weight standard in lane 1. Incubation of PSII submembrane fractions at low Al^3+^ concentrations (up to 3 mM), led to a loss of 17 and 23 kDa oxygen evolving extrinsic polypeptides, presented in lanes 3 and 4 as compared to the control in lane 2 (Fig. 3). However, as is distinctly seen, the band that represents the 33 kDa polypeptide was gradually reduced in intensity with increasing Al^3+^ concentrations. This polypeptide was partially removed at low concentrations of Al^3+^. Nevertheless, addition of higher concentrations of Al^3+^ (above 3 mM Al^3+^) caused a dissociation of the 17, 23 and 33 kDa polypeptides associated with the OEC (Fig. 3, lanes 5, 6). Based on the above data we note a correlation between the removal of extrinsic polypeptides associated with the OEC and the loss of oxygen evolution activity observed in Fig. 1 and consequently the destabilization of OEC of PSII complex treated with Al^3+^ concentrations. It is important to note that other polypeptides of PSII complex such as proteins of LHCII antenna remained bound to the PSII core and are not removed or degraded.

### Chl fluorescence induction

**Fig 4:**
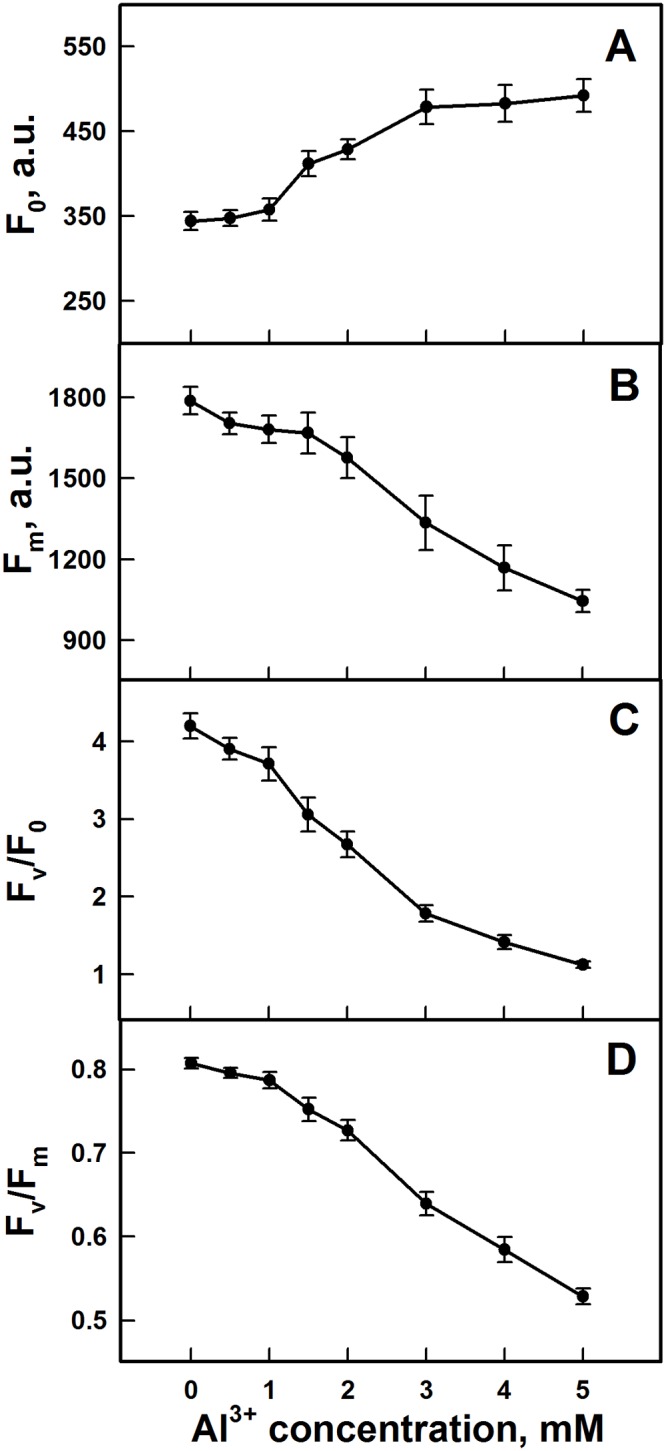
Effect of the addition of various Al^3+^ concentrations in PSII submembrane fractions on the Chl fluorescence parameters. **(A)** F_0_; **(B)** F_m_; **(C)** F_v_/F_0_ and **(D)** F_v_/F_m_. The data are the mean ± SD of nine independent measurements.

In order to evaluate the effects of Al^3+^ in the electron transport chain of PSII, chlorophyll fluorescence parameters of PSII submembrane fractions are measured. Fig. 4 shows the variation of F_0_, the initial Chl fluorescence obtained in dark adapted samples, F_m_, the maximal Chl fluorescence measured under saturating red-light illumination, the F_v_/F_0_ and F_v_/F_m_ ratios. Result in Fig. 4A shows an increase in F_0_ with increasing Al^3+^ concentrations. This effect was mainly marked above 2 mM. Nevertheless, with the same range of Al^3+^ concentrations, F_m_ registered a decline and reaches a larger decrease at higher levels of concentrations (above 2 mM) (Fig. 4B). The decline in F_m_ and the increase of F_0_ coincided with a strong decrease in both F_v_/F_0_, a parameter that accounts for the simultaneous variations in F_m_ and F_0_ in determinations of the maximum quantum yields of PSII [70], and the maximal quantum yield of PSII (F_v_/F_m_) (Figs. 4C, 4D). Addition of low concentrations of Al^3+^ (below 2 mM) to the submembrane fractions of PSII did not affect significantly the values of the maximal PSII photochemical quantum yield, F_v_/F_m_. However, at the same range of Al^3+^ concentrations, F_v_/F_0_ showed a significant decrease. In addition, F_v_/F_m_ and F_v_/F_0_ had obvious decreases with increasing concentrations of Al^3+^ above 2 mM, which can respectively reach 35% and 74% of reduction at 5 mM Al^3+^ compared to the control. This drop in F_v_/F_0_ and F_v_/F_m_ ratios observed with Al^3+^ concentrations correlates with the inhibition of oxygen evolution and the removal of the three extrinsic polypeptides associated with the OEC illustrated in Figs. 1 and 3, respectively.

**Fig 5:**
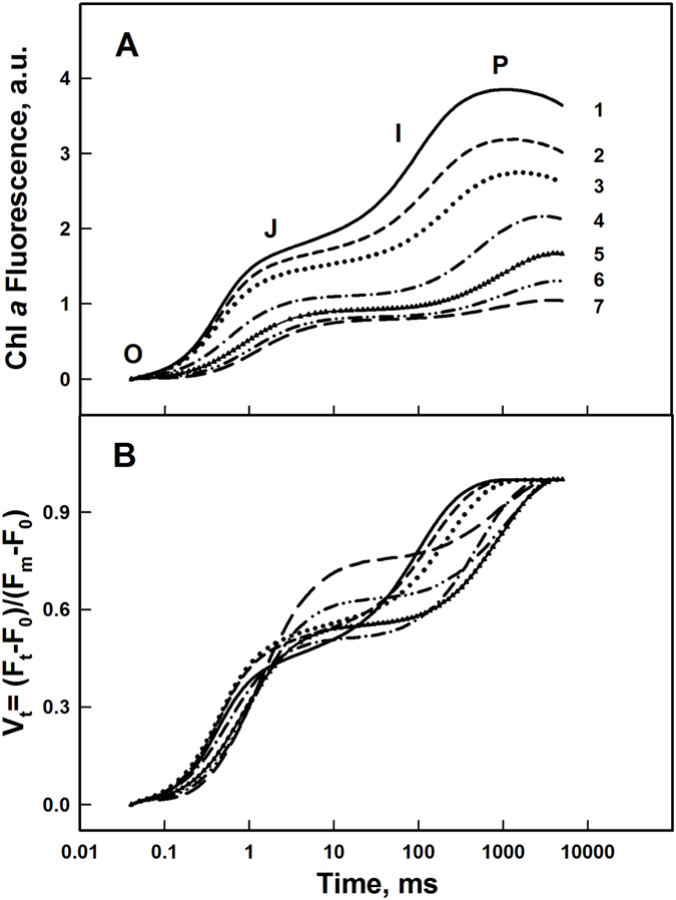
OJIP traces of Chl Fluorescence induction. **(A) of PSII submembrane fractions treated with different concentrations of Al^3+^**. (1) Control, (2) 1 mM, (3) 1.5 mM, (4) 2 mM, (5) 3 mM, (6) 4 mM, (7) 5 mM; **(B) normalized at both initial and maximal intensities**. Each curve is the average of nine independent measurements. See details in the Materials and Methods.

The OJIP traces of Chl fluorescence induction were obtained in order to elucidate the effect of Al^3+^ on the PSII photochemistry by characterizing the electron transport in both donor and acceptor sides of PSII [71]. The OJIP trace represents the successive reduction of the quinones located at the acceptor side of PSII [72] and it is composed of three main phases corresponding to OJ, JI, and IP [73–75] (Fig. 5A). OJ phase corresponds to the first phase and reflects the reduction of Q_A_, the primary quinone electron acceptor of PSII. The second phase (JI) reflects an accumulation of the Q_A_-Q_B_- form. Whereas, the last phase IP reflects the reduction of the plastoquinone pool together with reduction of the secondary quinone acceptor Q_B_ [58, 74, 75].

Fig. 5A shows that with increasing the Al^3+^ concentration, the yield of the OJIP curves was considerably decreased. This progressive decrease demonstrates a distinct reduction of the PSII capacity for electron transport from OEC toward quinone acceptors. This fact is well illustrated in Fig. 5B where the FI traces are normalized at both minimal and maximal values (Vt curves). Relative fluorescence intensity at OJ phase gradually increased with low Al^3+^ concentrations (below 2 mM). This suggests that the rate of Q_A_- reoxidation by Q_B_ was delayed [42, 76–78]. When Al^3+^ concentrations increased above 2 mM, the FI was damped progressively at all phases suggesting that the destabilized OEC becomes unable to supply electrons for PSII to reduce adequately the quinone acceptors of PSII thus decreasing the maximal fluorescence yield.

### Low temperature (77 K) Chl fluorescence emission spectra

**Fig 6:**
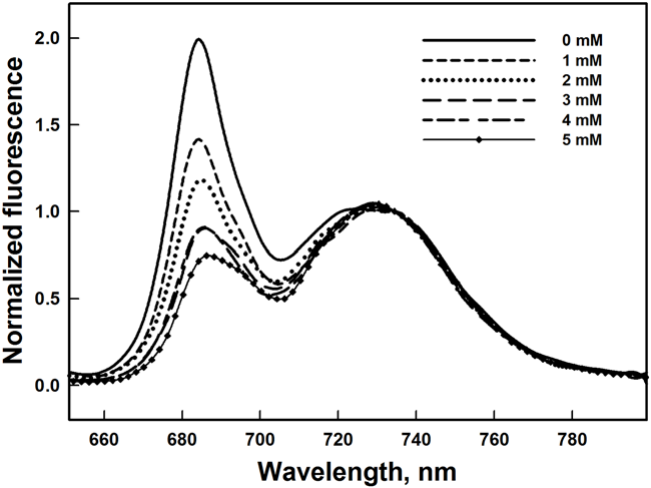
Changes in the Low temperature (77 K) chlorophyll fluorescence emission spectra of thylakoids membranes exposed to different Al^3+^ concentrations. The slit widths for excitation and emission were set at 5 and 2.5 nm, respectively. Spectra were normalized at 732 nm. The presented spectra are representative of three separate experiments.

In order to investigate the effect of Al^3+^ on the functional connection of the LHCII antenna to the PSII RC and therefore evaluate the excitation energy transfer to the PSII RC, we examined the changes in the 77 K Chl fluorescence emission spectra in isolated thylakoid membranes in the presence of Al^3+^ at various concentrations. At low temperature (77 K) the chlorophyll fluorescence emission spectra of the control thylakoid membranes exhibited the characteristic emission bands at 684, 692 and 732 nm. The emission band at 684 with shoulder at 692 nm is associated with the Chl *a* of PSII and the prominent band at 732 nm characterises the Chl *a* related to PSI [79–81]. Fig. 6 shows Chl fluorescence emission spectra obtained with excitation at 436 nm and also normalized at 732 nm. The relative amplitude of the peak at 684 nm gradually decreased with increasing Al^3+^ concentration. This effect was more pronounced at higher Al^3+^ concentrations. This result can indicate an alteration of LHCII, and consequently a decrease of excitation energy transfer from LHCII to PSII RC.

### Native green gel electrophoresis

**Fig 7:**
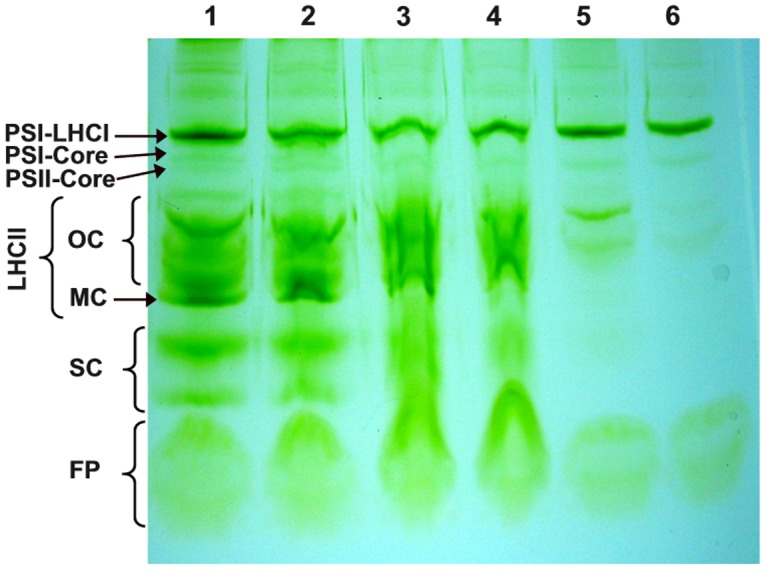
Native green gel electrophoresis of chlorophyll-protein complexes isolated from spinach thylakoid membranes treated with Al^3+^ at various concentrations. Lane 1: Control; lane 2: 1 mM Al^3+^ treated thylakoid membranes; lane 3: 2 mM Al^3+^ treated thylakoid membranes; lane 4: 3 mM Al^3+^ treated thylakoid membranes; lane 5: 4 mM Al^3+^ treated thylakoid membranes; lane 6: 5 mM Al^3+^ treated thylakoid membranes. Symbols on the left side indicate the following separated bands: PSI-LHCI, a number of large PSI complexes with attached LHCI antenna; PSI core, core protein complexes of PSI; PSII core, core protein complexes of PSII; LHCII OC, oligomeric LHCII complexes; LHCII MC, monomeric LHCII complexes; SC, small complexes; FP, free pigments. The electrophoretic patterns are representative for three independent experiments.

To gain deeper insight into effect of Al^3+^ addition on the chlorophyll-protein complexes, green gel electrophoresis of isolated thylakoid membranes treated with different concentrations of Al^3+^ was performed (Fig. 7). As shown in Fig. 7, lane 1, we observed five major subunits of chlorophyll-protein complexes in the control of isolated thylakoid membranes: RC PSI-LHCI, PSI core (core protein complexes of PSI), PSII core (core protein complexes of PSII), LHCII (oligomers and monomers), small complexes (SC) and free pigments (FP) [82]. This lane was used as a standard of the gel electrophoretic analysis of isolated thylakoid membranes treated with different concentrations of Al^3+^. The addition of 1–5 mM Al^3+^ in isolated thylakoid membranes resulted in a gradual change in the electrophoretic pattern of the chlorophyll-protein complexes. Addition of Al^3+^ at low concentrations (below 3 mM), induced especially a disturbance of the LHCII complex (Fig. 7, lane 3). This effect was observed at concentrations of 2 and 3 mM Al^3+^, and also affected other bands such as SC and FP (Fig. 7, lanes 3, 4). Notably, at high concentrations of Al^3+^ (above 3 mM) we observed the disappearance of the chlorophylls of both LHCII and SC complexes bands. This may be due essentially to an important loss of the chlorophylls of LHCII and SC complexes bands (Fig. 7, lanes 5, 6). In addition, results shown by SDS-gel electrophoresis demonstrates that antenna proteins of LHCII complex remained bound to the PSII core and are not removed which minimize the possibility of their degradation (Fig. 3). Nevertheless, the LHCI complex band seemed not to be affected by the concentrations of Al^3+^. Therefore, the alteration of the native structures of these chlorophyll-protein complexes of PSII, especially LHCII complex, were correlated with many parameters such as, the drop in fluorescence intensity in emission peaks at 684 nm at low temperature (77 K), the increase of both F_0_ and misses percentage. Thus, these changes resulted in a disturbance of the energy transfer from LHCII to PSII RC.

### FTIR spectra of Al^3+^-PSII complexes

**Fig 8:**
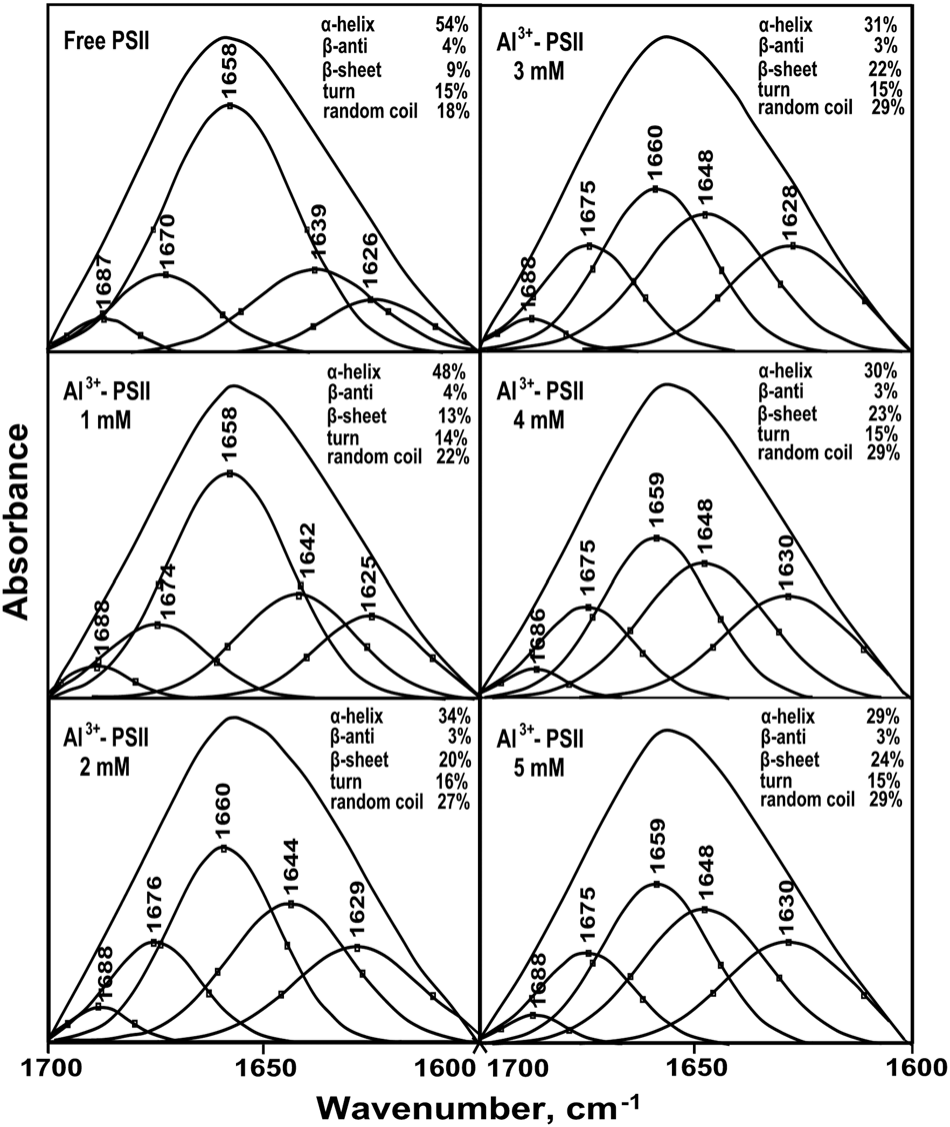
Second derivative resolution enhancement and curve-fitted amide I region (1700–1600 cm^-1^) of IR spectra of PSII proteins in the presence of Al^3+^ at various concentrations. The presented spectra are representative of three separate experiments.

**Fig 9:**
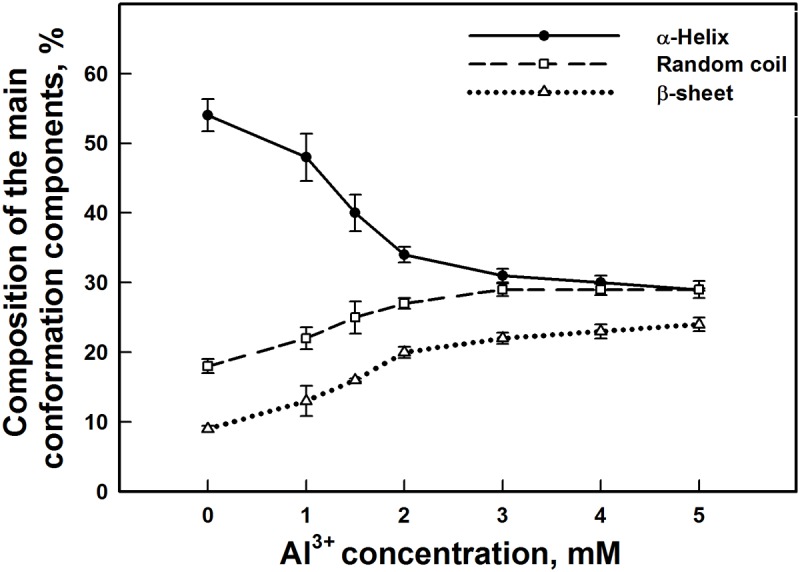
Variation of the composition of the main conformation components (%) of PSII proteins in spinach PSII submembrane fractions treated with various Al^3+^ concentrations. The data are the mean ± SD of three independent measurements.

FTIR spectroscopy is a very powerful technique applied to investigate the secondary structure of several proteins, such as soluble and membrane proteins. Also, it has been used to determine the secondary structure of protein complexes having a structural complexity and with high molecular weight such as protein complexes of PSII [83–86]. The infrared amide I band in the 1700–1600 cm^-1^ region shows a strong absorption at 1658 cm^-1^ that originates from the C = O stretching vibrational mode in the peptide group [87, 88]. This band is sensitive to the protein complexation and changes in the secondary structure and it is widely used for studying protein conformation [86, 89]. In our present study, we have used FTIR spectroscopy in order to provide more detailed information about changes in the secondary structure of the proteins of PSII submembrane fractions induced by addition of Al^3+^. A quantitative secondary structure analysis using the infrared absorption spectra and decomposition of amide I band of the free PSII proteins and their Al^3+^ complexes with various concentrations are performed and the results are presented in Figs. 8 and 9. Based on the curve-fitting analysis method, the secondary structure of the free PSII protein complexes contained 54% α-helix (1658 cm^-1^), 9% β-sheet (1626 cm^-1^), 15% turn structure (1670 cm^-1^), 4% β-antiparallel (1687 cm^-1^) and 18% random coil (1639 cm^-1^). The addition of Al^3+^ at low concentrations (below 2 mM), induced a change in the secondary structure of the PSII proteins due to the formation of an Al^3+^-PSII complex. This change was shown by a decrease of α-helix and an increase of β-sheet and random coil structures while the turn and β-antiparallel structures remained steady. With increasing Al^3+^ concentration above 2 mM, major changes of some conformational components were observed. Compared to free PSII proteins, the secondary structure of the Al^3+^-complex at 5 mM showed a significant decrease of the α-helix content from 54% to 29%, accompanied by an important increase of the β-sheet and random coil contents from 9% to 24% and 18% to 29%, respectively. Nevertheless, both the turn and β-antiparallel structures were still stable at the presence of Al^3+^ even at high concentrations (Figs. 8, 9). This important change of protein secondary structure in PSII complex in the presence of Al^3+^ demonstrates conformational changes of PSII proteins, which may be due to the denaturation of these proteins of PSII complex that affected PSII function.

## Discussion and Conclusions

In this study, we used isolated PSII submembrane fractions to investigate in detail the modes and the mechanisms of the inhibitory action of Al^3+^ in PSII complex by analyzing the interaction of Al^3+^ with protein subunits of PSII. It has been shown that Al^3+^ exerts an inhibitory effect on PSII activity at concentrations of millimolar range [42]. In fact, our results confirm the decrease of the oxygen evolution activity by the addition of 2–3 mM of Al^3+^ (Fig. 1). This inhibition of oxygen yield was closely related to the interaction of Al^3+^ with the donor side of the PSII causing the release of the two extrinsic polypeptides of 17 and 23 kDa associated with the OEC at the luminal side (Fig. 3) [17, 25, 26]. However, the latest extrinsic polypeptide of 33 kDa associated with the OEC remained bound to PSII and not much affected by the presence of Al^3+^ at these concentrations (Fig. 3). In addition, previous studies have demonstrated that the release of these two extrinsic polypeptides of 17 and 23 kDa reduces the binding affinity of the cofactors, such as Ca^2+^ and Cl^−^, for the OEC [17]. Also, several studies revealed that these cofactors (Ca^2+^ and Cl^−^) are important to maintain the active conformation of the OEC keeping the proper advancement of the S states of the Mn_4_O_5_Ca cluster [41, 90, 91].

Under 2–3 mM of Al^3+^ treatment, the advancement of the S states of the Mn_4_O_5_Ca cluster associated with the OEC was only slightly affected (Fig. 2). This could be explained by the presence of the extrinsic polypeptides of 33 kDa which allows the stabilization of the Mn_4_O_5_Ca cluster and modulates the Ca^2+^ and Cl^-^requirements for oxygen evolution [18]. Moreover, it has been shown that the absence of extrinsic polypeptides of 33 kDa induces the release of two or four Mn ions leading to the loss of the oxygen evolving activity [92]. At the same lowest Al^3+^ concentrations (below 3 mM), a relative increase of the OJ rise in the OJIP curves was shown in Fig. 4B, indicating a delay in reoxidation of the primary PSII quinone acceptor Q_A_-. This fact has been confirmed by Hasni et al. (2013) [42] in thylakoid membranes treated with concentrations of Al^3+^ below 3 mM. Indeed, it has been shown also that the removal of 17 and 24 kDa extrinsic polypeptides and/or Ca^2+^ induces the modification in the Q_A_ mid-point potential, increasing the life-time for Q_A_- reoxidation [93, 94].

However, at high Al^3+^ concentrations, especially at 5 mM, oxygen yield was completely abolished. In addition, this effect was accompanied with a drastic decline in the amplitudes of the flash-induced oxygen yields and a loss of characteristic oscillations (Fig. 2). This change in the flash pattern might be explained by a severe reduction and/or destruction of the total number of functionally active oxygen evolving centers [77, 78]. This indicates that the OEC was severely impaired. This impairment was associated, in part, with the release of the three extrinsic polypeptides of 17, 23 and 33 kDa (Fig. 3). Similar results were obtained with several cations metal such as Cd^2+^, Cu^2+^, Hg^2+^, Ni^2+^, Pb^2+^ and Zn^2+^ [37, 39, 41, 52, 53, 95, 96].

On the other hand, the pattern of oxygen evolution showed a rise in the values of misses and of double hits (Table 1), suggesting that higher concentrations of Al^3+^ could affect directly the Mn_4_O_5_Ca cluster and induce a delay of the transition between the S states [97]. This may be due to the loss of Ca^2+^ and/or Mn from Mn_4_O_5_Ca cluster. Therefore, the cluster is disorganized and becomes not functional. This effect was associated with the removal of the extrinsic polypeptides of 33 kD from the PSII complex, which is considered as an important protein for the functional conformation of the catalytic Mn_4_O_5_Ca cluster [98]. In addition, Miyao et al. (1987) [24] have demonstrated that the depletion of PsbO retards significantly the transition between S_3_-[S_4_] →S_0_.

Shevela et al. (2006) [99] have proposed that the addition of bicarbonate causes a delay in the transition between S states of Mn_4_O_5_Ca cluster. This delay is attributed to the binding reaction of bicarbonate with Mn ions causing changes in the redox properties of the OEC. Further, PospÌail et al. (2003) [100] and Barra et al. (2005) [101] have claimed that the inactivation of OEC by heat stress is related to the release of extrinsic proteins from the thylakoid membrane, followed by progressive release of Mn atoms. In concordance with our results, Sandusky and Yocum (1986) [90] have reported that some monoamines induce an inhibition of oxygen evolution activity and affect the distribution of higher S states of the Mn_4_O_5_Ca cluster. Our results are in agreement with several works which demonstrated that addition of Cu^2+^ affects both the Mn_4_O_5_Ca cluster and the extrinsic proteins of the OEC at the donor side of PSII [53, 102–105]. At 5mM of Al^3+^, the complete loss of oxygen yield was accompanied by a total inhibition of electron transfer from donor side to acceptor side of PSII. This was shown by a strong damping of the Chl FI and was also reflected by a drastic decrease in F_v_/F_0_ and F_v_/F_m_ ratios (Fig. 4A, 5C, 5D). In a recent paper, Hasni et al. (2013) [42] have demonstrated that the decline in F_v_/F_m_ together with the amplitudes of the OJ and IP phases of the OJIP traces, in the presence of Al^3+^ in thylakoid membranes, was associated with the impairment of OEC, reducing the electrons transfer to PSII and consequently the decrease in the maximal fluorescence yield. In addition, Hasni et al. (2013) [42] have demonstrated that the disorganization of the OEC by Al^3+^, at concentration above 3 mM, causes an alteration of the molecular surrounding of Tyr Z leading to the inhibition of electron transfer from Tyr Z to P680^+^. Consequently, the inhibition of electron donation to PSII has promoted an accumulation of P680^+^ species quenching the fluorescence intensity of PS II at the peak of 684 nm (S1 Fig.).

This mechanism has been discussed in detail as described in Hasni et al. (2013) [42]. Similar results have been recently reported in pea thylakoid membranes treated with UV-B irradiation [106]. Further, our results strongly support the reports of Msilini et al. (2011) [77], Hamdani and Carpentier (2009) [78] and Ait Ali et al. (2006) [107] suggesting that the inhibition of the oxygen yields together with the damping of Chl fluorescence may be caused by a reduced number of active PSII RC.

It is important to note that F_0_ increased with increasing Al^3+^ concentrations. This increase may be due to the reduction in the energy transfer from the antennae complexes to the RC of PSII [78, 108–110]. This suggests that the release of the extrinsic polypeptides facilitates the interaction of Al^3+^ with the antennae complexes at the luminal side of PSII resulting in an increase in F_0_. This result is corroborated by an increase in the percent of misses and the drop of the peak at 684 nm of the fluorescence intensity emission at low temperature (77 K) (Table 1, Fig. 6). In addition, the results, provided by the native green gel electrophoresis and SDS-gel electrophoresis, reflected changes in the structure of the architecture of LHCII (Figs. 3 and 7). In concordance with our data, similar results have been shown under Cd and Cu effects [31, 32, 54, 111, 112]. These changes are probably attributed to the denaturation of the LHCII antenna proteins accompanied by the loss or degradation of the chlorophylls pigment under Al^3+^ treatment (Figs. 3 and 7). Kochubey (2010) [113] demonstrated that heat stress causes damage in the protein conformation, changing the ensemble structure of chlorophyll and leading to disrupt energy transfer from the antennae complexes to the RC of PSII. In line with these results, Hamdani and Carpentier (2009) [78] have also suggested that the interaction of methylamine with the amino acids of the large hydrophilic loops of the proximal antenna protein of CP47 and/or CP43 induces a conformational change leading to the inhibition of the transfer of excitation energy from these complexes to the RC. In addition, Roose et al. (2007) [19] and Boekema et al. (2000) [36] have suggested that the release of the extrinsic proteins associated with the OEC may affect the intrinsic core components of PSII. Moreover, the removal of the two extrinsic polypeptides PsbP and PsbQ (of 23 and 17 kDa) has been proposed to affect the position of peripheral antenna proteins. Roose et al. (2010) [29] have suggested that the removal of the PsbP and PsbQ extrinsic polypeptides may induce transmembrane alterations in the structure of PSII complex leading to disruption of the Q_A_ and/or Q_B_ sites or modification of the plastoquinone-plastoquinol exchange channel. Besides that, the removal of the third extrinsic polypeptides PsbO (of 33 kDa) might induce additional changes in the positions of the peripheral antenna proteins and causes a destabilization in the dimeric structure of PSII leading to the conformational changes [19, 36].

Therefore, the results described above demonstrated that the functional alteration of PSII activity by Al^3+^ effect should be closely related with structural changes within PSII complex. To provide more detailed information about the changes of the secondary structure content of PSII complex in the presence of Al^3+^ concentrations, we used FTIR spectroscopy. With increasing Al^3+^ concentrations, the main conformational components obviously changed. This was caused probably by electrostatic interaction between positive charges of Al^3+^ with protein groups leading to local perturbations of protein structure [57]. These conformational changes are shown by a major secondary structural alterations reflected by the decrease of the α-helix, and an increase of the β-sheet and random-coil structures, while no major alterations were observed for the β-anti and turn structures (Figs. 8 and 9). This suggests that the α-helix structure is modified simultaneously with the β-sheet and random-coil structures. Therefore, this implies that a major component of PSII complex was denatured. Our result also supports the finding of Nahar et al. (1997) [57] and Nahar and Tajmir-Riahi (1996) [114] claiming the interaction of some divalent and trivalent cations metal such as Hg^2+^, Cd^2+^, Pb^2+^, Ga^3+^ and Al^3+^ with proteins of PSII complex. According to these studies, analysis of FTIR amide I band in the presence of these metal cations has showed especially, a decrease of α-helix structures and an increase of β-sheet contents. Besides that, an increase of random-coil structures was shown only under Al^3+^ and Ga^3+^ treatments. These alterations in the secondary structures led to major conformational changes of PSII proteins [57, 114].

It is important to note that, in the presence of high Al^3+^concentrations, a clear relationship was observed between the loss of oxygen evolution activity, inhibition of energy transfer from antennae complexes to RC on PSII and the modification of the protein secondary structures. We propose that Al^3+^ may disrupt polypeptides secondary structure in PSII enriched submembrane fractions at high concentrations, causing conformational changes of transmembrane intrinsic polypeptides rich in α-helices such as D1, D2. CP43, CP47, LHCII and other intrinsic polypeptides associated with both the OEC and the Q_B_ niche [3, 31, 32]. This modification leads to reduction in the percentage of α-helix structures. Moreover, we suggest that the removal of the three extrinsic polypeptides of 17, 23 and 33 kDa may promote an increase in the percentage of random-coil contents at the luminal side of PSII complex. This modification is accompanied with a complete loss of oxygen evolution activity. At the same range of Al^3+^ concentration, we have observed a change in the LHCII structure, showed by an increase of F_0_ and the percent of misses, in parallel with a decrease in the percentage of α-helix structures and an increase of β-sheet contents leading to inhibition in the energy transfer from LHCII to RC of PSII. All these transmembrane conformational changes probably modify the mid-point potential of both Q_A_ and Q_B_ and consequently cause an impairment of the quinone reduction on the acceptor side of PSII, resulting the inhibition of electron transfer and consequently the drop in PSII activity.

To our knowledge, our study is the first detailed work that provides structural data about the interaction mechanism of Al^3+^ with the PSII complex. The set of results demonstrate that the interaction of Al^3+^ with the intrinsic and extrinsic polypeptides of PSII complex induces major alterations of the protein secondary structure leading to conformational changes. These structural changes are closely related with the functional alteration of PSII activity. At the donor side of PSII, these changes cause the release of extrinsic polypeptides and disorganize the Mn_4_O_5_Ca cluster resulting in impairment of the OEC which leads to a complete loss of the oxygen evolution activity. Indeed, conformational changes of transmembrane intrinsic polypeptides cause especially an alteration of LHCII, reducing the energy transfer to PSII RC. Therefore, this disruption in polypeptides secondary structure of PSII probably affect other intrinsic polypeptides causing an impairment of the quinone reduction on the acceptor side of PSII leading to inhibition of electron transport activity.

## Supporting Information

S1 Fig Fluorescence emission spectra at room temperature of isolated thylakoid membranes treated with different Al^3+^ concentrations. The slit widths for excitation and emission were set at 5 and 2.5 nm, respectively. The Chl content of the samples was adjusted to 5 μg.ml^-1^. The presented spectra are representative of three separate experiments.
(TIF)
