# Supplementary figures and images for: Mechanism of Interaction of Al3+ with the Proteins Composition of Photosystem II
Source: PLoS One. 2015 Mar 25;10(3):e0120876. doi: 10.1371/journal.pone.0120876 (PMC4373732; doi:10.1371/journal.pone.0120876)

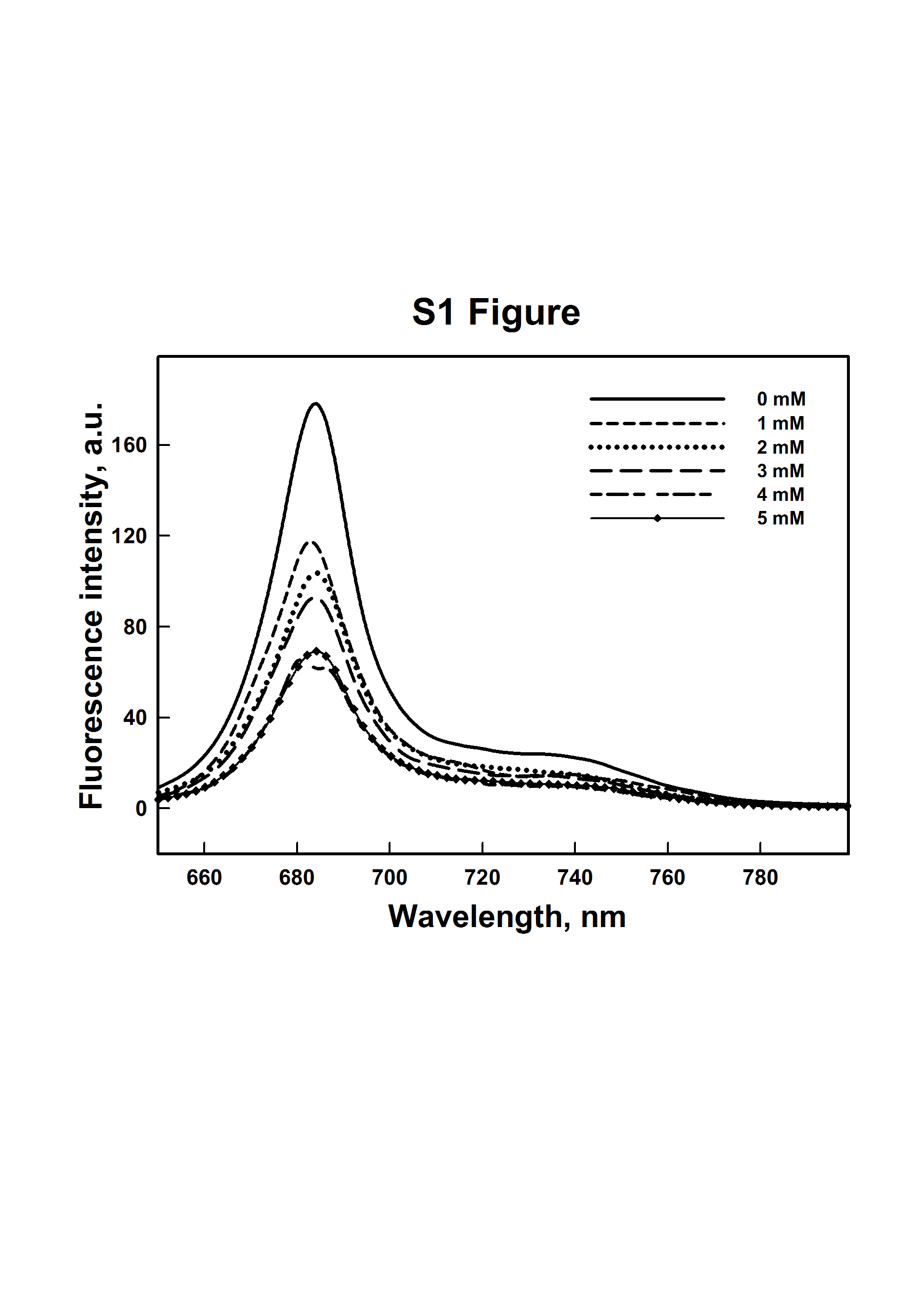

Supplement: S1 Fig — The slit widths for excitation and emission were set at 5 and 2.5 nm, respectively. The Chl content of the samples was adjusted to 5 μg.ml-1. The presented spectra are representative of three separate experiments. (TIF) [file pone.0120876.s001.tif]
